# Attempts to quit smoking, use of smoking cessation methods, and associated characteristics among COPD patients

**DOI:** 10.1038/s41533-022-00316-5

**Published:** 2022-11-10

**Authors:** Yekaterina Pashutina, Daniel Kotz, Sabrina Kastaun

**Affiliations:** 1 Institute of General Practice (ifam), Centre for Health and Society (chs), Addiction Research and Clinical Epidemiology Unit, Medical Faculty, Heinrich‐Heine‐University Duesseldorf, Duesseldorf, Germany; 2 Usher Institute of Population Health Sciences and Informatics, University of Edinburgh, Edinburgh, United Kingdom; 3 Institute of General Practice (ifam), Centre for Health and Society (chs), Patient-Physician-Communication Research Unit, Medical Faculty, Heinrich‐Heine‐University Duesseldorf, Duesseldorf, Germany

**Keywords:** Chronic obstructive pulmonary disease, Epidemiology

## Abstract

We explored past-year quit attempts, cessation methods used, and associations with sociodemographic, smoking, and health-related characteristics among smoking patients with chronic obstructive pulmonary disease (COPD) in Germany. Cross-sectional survey data of 509 past-year smokers (current smokers and ≤12 months abstinent) with COPD (ICD-10 code J44.x and FEV1/FVC <0.70) from 19 pulmonary primary care practices were used. Associations were explored between age, sex, educational qualification, lung function, urges to smoke, psychological distress, and (a) ≥1 past-year quit attempt (yes/no), (b) use of ≥1 evidence-based smoking cessation method (yes/no). Of all patients, 48.5% (*n* = 247, 95% confidence interval (CI) 44.2–52.9) reported ≥1 past-year quit attempt. Such an attempt was positively associated with the male sex (Odds Ratio (OR) = 1.50, 95% CI 1.01–2.24) and negatively associated with time spent with urges to smoke (OR = 0.69, 95% CI 0.52–0.91). During the most recent past-year quit attempt, one-third of the patients used ≥1 evidence-based smoking cessation method (31.2%, 95% CI 25.4–37.0), which was positively associated with the strength of urges to smoke (OR = 1.62, 95% CI 1.09–2.41). Combined behavioural and pharmacological treatments were used by 4.0% (*n* = 10, 95% CI 1.6–6.5). Electronic cigarettes were used most frequently (21.5%, 95% CI 16.3–26.6). Although a high proportion of COPD patients in German pulmonary primary care attempt to quit smoking, only a few of them use evidence-based methods as assistance for quitting.

## Introduction

Tobacco smoking is the leading risk factor for the development of chronic obstructive pulmonary disease (COPD)^1^, and continued smoking can accelerate COPD progression^2^. Among patients with COPD, smoking cessation is the most effective treatment that reduces the excessive decline in lung function^3–5^, improves respiratory symptoms^5,6^, decrease the risk of exacerbations^7^ and hospital admission^8^, and improves survival rates^3,9,10^. Nevertheless, a previous German cohort study showed a high smoking prevalence of 38% among COPD patients^11^.

Tobacco dependence and psychological distress influence persistent tobacco consumption^12^ and affect quitting behaviour^13,14^. In turn, smokers with COPD report higher levels of tobacco dependence than smokers without COPD^15–17^. Anxiety and depression are common comorbidities of COPD^18^, and COPD patients experience more psychological distress compared to the general population^19^. As a result, quitting smoking seems to be more difficult for smokers with COPD than for healthy smokers^16^.

According to international research, between 48–65% of smokers with COPD attempted to quit smoking in the past year^20–23^. Quit attempts were positively associated with younger age^21^, female gender^22^, and higher educational qualification^23^. To our knowledge, data on quit attempts and on associated characteristics among patients with COPD in Germany are missing.

To support quitting smoking, the German guidelines for the diagnosis and treatment of COPD recommend various evidence-based methods, including pharmacological (e.g., nicotine replacement therapy (NRT), varenicline, bupropion) and behavioural (e.g., brief physician advice, individual, group or telephone counselling) treatments, which increase the chances of successful long-term abstinence compared with unassisted quitting^24,25^. The guidelines especially recommend the combination of behavioural and pharmacological treatments as the most effective approach to assist smoking cessation^24,25^. Alternative methods, such as electronic cigarette (EC), acupuncture, or hypnotherapy, currently have no clarified evidence and therefore find no recommendation in the guidelines^24,25^. A former cross-sectional, web-based survey on smokers with lung conditions (70% of them with COPD) across Europe showed that, in this population, the most frequently used method to support smoking cessation was NRT (31%), followed by EC (20%)^26^. Usage rates of other evidence-based and alternative methods were reported as follows: evidence-based varenicline (13%), bupropion (9%), and telephone quitlines (3%), and alternative methods such as acupuncture (7%) and hypnotherapy (5%)^26^. In Germany, there is a lack of information on the use of evidence-based and alternative smoking cessation methods among smokers with COPD, and it remains unclear whether sociodemographic, smoking, and health-related characteristics are associated with the use of evidence-based cessation methods in this smoking population.

Therefore, among a clinical sample of the adult (≥18 years) current smokers and recent ex-smokers (≤12 months since quitting) with clinically diagnosed COPD, we aimed to estimate:

1. the prevalence of self‐reported past-year quit attempts, and to characterise these quit attempts (i.e. number of attempts, whether the most recent attempt was made abruptly or by cutting down first, and whether it was planned or unplanned);
2. the prevalence of the use of evidence-based and alternative methods to assist the most recent past-year quit attempt;
3. associations between sociodemographic characteristics, percentage of predicted forced expiratory volume in 1 s (FEV1% predicted) as a parameter of COPD severity^1^, time spent with urges to smoke, the strength of urges to smoke, psychological distress, and the presence of ≥1 self‐reported past-year quit attempt;
4. associations between sociodemographic characteristics, FEV1% predicted, time spent with urges to smoke, the strength of urges to smoke, psychological distress, and the use of ≥1 evidence-based method to assist the most recent past-year quit attempt.

## Methods

### Design, setting and participants

We used data from the ‘Use and real-world effectiveness of smoking cessation methods in patients with COPD’ (RESPIRO) study. This cross-sectional survey among COPD patients was conducted in 19 pulmonary practices across the German federal state of North Rhine-Westphalia (NRW) between September 2018 and June 2020. The RESPIRO study was prospectively registered at the German Clinical Trials Register (DRKS00011322) and approved by the ethics committee of the Medical Faculty of the Heinrich-Heine-University Duesseldorf, Germany (ID 5680 R).

Pulmonary practices were recruited through the scientific information network of the Scientific Institute for Health Care Research in Pneumology ‘WINPNEU’ (https://winpneu.de), initiated by the German federal association of pulmonologists, sleep and respiratory physicians. A contact person within the practice (i.e. the pulmonologist, or a study nurse) carried out the recruitment of patients aged ≥18 with the diagnosis of COPD according to the 10th revision of the International Statistical Classification of Diseases and Related Health Problems (ICD-10 code: J44.x)^27^. The contact person also documented the clinical characteristics of participating patients in the study questionnaire, including the ICD-10 code for COPD and lung function parameters of the most recent spirometry: forced expiratory volume in 1 s (FEV1), forced vital capacity (FVC), and FEV1% predicted. Eligible patients received all study materials (questionnaire, informed consent form, and a small non-financial incentive) to take home with them and were asked to send the completed documents back to the study centre.

Of 4377 distributed questionnaires, 2012 questionnaires were sent back (46%). Six responders withdrew their informed consent to participate afterwards. Among the remaining 2006 responders, 2004 responders had a valid ICD-10 code for COPD entered in their questionnaire. Of these, 653 (32.6%) had reported to be current smokers of cigarettes or other combustible tobacco products (e.g. pipe, cigars), 142 (7.1%) had reported to be recent ex-smokers (≤12 months since quitting), 957 (47.8%) had reported to be long-term ex-smokers (>12 months since quitting), and 162 (8.1%) had reported to be never smokers (4.5%, *n* = 90 no answer).

For the present study, we conservatively included only past-year smokers (current smokers and recent ex-smokers) with a post-bronchodilator ratio of FEV1/FVC < 0.70, which we calculated according to the Global Initiative for Chronic Obstructive Lung Disease (GOLD) criteria for the diagnosis of COPD^1^. Of all 795 past-year smokers listed as COPD patients in the practices according to ICD-10^27^, 286 (36%) had an FEV1/FVC ≥0.70 and were thus excluded from final statistical analyses. This resulted in a final study sample of 509 past-year smokers with a spirometry-confirmed COPD diagnosis.

### Outcome variables

Past-year quit attempts were measured by asking about serious attempts to stop smoking in the past year; full details are provided in Table 1. For statistical analyses, the number of attempts was recoded into a dichotomous variable ‘past-year quit attempt’ (yes, ≥1 attempt versus no attempt). To characterise these attempts, the absolute number of attempts was categorised into four groups: one attempt, two attempts, three attempts, and four or more attempts.

**Table 1:** Measures used to assess quit attempts, time spent with urges to smoke, the strength of urges to smoke and psychological distress.

Outcome measure:	Exposure measures:
Quit attempts	Time spent with urges to smoke and strength of urges to smoke (SUTS)
‘How many serious attempts to stop smoking have you made in the last 12 months? By serious attempt, I mean you decided that you would try to make sure you never smoked again. Please include any attempt that you are currently making and please include any successful attempt made within the last year.’	1) How much of the time have you felt the urge to smoke in the past 24 h? - not at all (score 0) - a little of the time (score 1) - some of the time (score 2) - a lot of the time (score 3) - almost all of the time (score 4) - all the time (score 5) 2) In general, how strong have the urges to smoke been? - slight (score 1) - moderate (score 2) - strong (score 3) - very strong (score 4) - extremely strong (score 5) The second item was coded ‘0’ for those respondents who answered ‘not at all’ on the first item.
	Psychological distress (PHQ-4)
	Over the last 2 weeks, how often have you been bothered by the following problems? 1) Little interest or pleasure in doing things 2) Feeling down, depressed, or hopeless 3) Feeling nervous, anxious or on edge 4) Not being able to stop or control worrying Response options were: ‘not at all’ (coded 0), ‘several days’ (coded 1), ‘more than half the days’ (coded 2) and ‘nearly every day’ (coded 3).

SUTS strength of urges to smoke scale^29^, *PHQ-4* patient health questionnaire-4^30,31^.

Furthermore, smokers with ≥1 past-year quit attempt were asked, whether their most recent quit attempt was made abruptly or by cutting down first, and whether it was planned or initiated spontaneously. This group was also asked about the use of evidence-based and alternative smoking cessation methods to assist their most recent past-year quit attempt (multiple answers were allowed). Evidence-based methods were chosen according to the current German guidelines for the diagnosis and treatment of COPD patients^24,25^: behavioural (brief physician advice, individual, group or telephone counselling) and pharmacological (NRT with/without a prescription, varenicline, and bupropion) methods. For regression analyses, we coded a new dichotomous variable, ‘use of ≥1 evidence-based smoking cessation method’ (yes versus no). Alternative methods were chosen according to frequently used methods in the German general smoking population^28^.

### Exposure variables

Sociodemographic characteristics measured were: age, sex (male, female), and educational qualification (low = 9 years of education or no graduation, medium = 10 years, high ≥11 years).

The FEV1% predicted was used to categorise COPD severity for descriptive statistics: FEV1 ≥80% predicted = mild (GOLD 1), 50% ≤FEV1 <80% predicted = moderate (GOLD 2), 30% ≤ FEV1 <50 % predicted = severe (GOLD 3), and FEV1 <30% predicted = very severe (GOLD 4) COPD^1^. For regression analyses, the FEV1% predicted was used as a continuous variable.

Time spent with urges to smoke and strength of urges to smoke were measured by using the German version of the Strength of Urges to Smoke Scale (SUTS)^29^ assessing tobacco dependence; full details are provided in Table 1. Both items were included as continuous variables (range 0 to 5) for regression analyses.

Psychological distress was measured using the validated, ultra-brief German version of the Patient Health Questionnaire-4 (PHQ-4)^30,31^ assessing symptoms of major depression and generalised anxiety; full details are provided in Table 1. The total PHQ-4 score ranges from 0 to 12, with a score of 6 or above representing psychological distress. For descriptive statistics, the PHQ-4 was used as a dichotomous variable by using this cut-off. For regression analyses, the PHQ-4 was used as a continuous variable.

### Statistical analyses

The study protocol and analysis plan were written prior to analysing data and pre‐registered on the Open Science Framework: https://osf.io/a24t3/. All statistical analyses were conducted using IBM SPSS Statistics Version 28.0.

To assess research aims 1 and 2, we used complete case data and presented prevalence data together with 95% confidence intervals (95% CI).

A multivariable logistic regression model was used to assess the association between age, sex, educational qualification, FEV1% predicted, time spent with urges to smoke, the strength of urges to smoke, psychological distress and the dichotomous outcome ‘past-year quit attempt’ (1 = yes, ≥1 attempt versus 0 = none) among all 509 past-year smokers (research aim 3). Among past-year smokers with ≥1 quit attempt, we repeated this analysis with the dichotomous outcome ‘use of ≥1 evidence-based smoking cessation method’ (1 = yes versus 0 = none) during the most recent past-year attempt (research aim 4).

Since questionnaires were self-completed, missing data was relatively high and occurred in the total study sample in educational qualification (10.0%, *n* = 51), time spent with urges to smoke (5.1%, *n* = 26), the strength of urges to smoke (4.9%, *n* = 25), psychological distress (9.4%, *n* = 48), and past-year quit attempt (13.2%, *n* = 67). Therefore, we used multiple imputations to impute missing data of all variables of interest included in regression models. Imputations were based on logistic regression models (for dichotomous and categorical variables) and predictive mean matching (for continuous variables) using the multivariate imputation by chained equations (MICE) algorithm^32^. Ten imputed datasets with ten iterations per dataset were created^33^. Results of analyses across the imputed datasets were combined using Rubin’s rules^34^ and presented as odds ratio (OR) with 95% CI and *p* value.

Regressions were repeated in the total sample of all 795 past-year smokers listed as COPD patients in the practices according to ICD-10^27^ (including those with an FEV1/FVC ≥0.70 calculated by us) (Supplementary Table 1).

### Ancillary analysis

In the sample of current smokers with COPD (FEV1/FVC < 0.70), we conducted a post-hoc descriptive analysis of a current motivation to stop smoking, measured using the validated German version of the Motivation To Stop Scale (MTSS)^35,36^ (Supplementary Table 2).

### Reporting summary

Further information on research design is available in the Nature Research Reporting Summary linked to this article.

## Results

Sociodemographic, smoking, and health-related characteristics of the study sample are presented in Table 2. The patients had a mean age of 62.8 years (standard deviation (SD) = 8.4 years, range 35–87 years), and 56.2% of them were male.

**Table 2:** Sociodemographic, smoking, and health-related characteristics of 509 past-year smokers with COPD (FEV1/FVC < 0.7).

	Total % (*n*)
Age (years), mean (SD)	62.8 (8.4)
Sex	
Female	43.8 (223)
Male	56.2 (286)
Educational qualification^a^	
Low	55.8 (284)
Medium	22.6 (115)
High	11.6 (59)
Smoking status	
Current smokers	81.7 (416)
Recent ex-smokers (<12 since quitting)	18.3 (93)
Quit attempts	
Yes, 1 or more	48.5 (247)
No	38.3 (195)
Time spent with urges to smoke, mean (SD)^b^	2.5 (1.3)
Strength of urges to smoke, mean (SD)^b^	2.2 (1.2)
Psychological distress^c^	
Yes ≥6	22.2 (113)
No <6	68.4 (348)
Severity of COPD^d^	
GOLD 1	2.9 (15)
GOLD 2	44.6 (227)
GOLD 3	40.3 (205)
GOLD 4	12.2 (62)

Data were presented as percentages (number, *n*), unless stated otherwise. SD standard deviation. Differences when calculating the total percentage can be explained by missing data on the respective variable.

^a^German educational qualification: low = 9 years of education or no graduation, medium = 10 years of education, high = ≥11 years of education.

^b^Both items were measured with the German version of the Strength of Urges to Smoke Scale (SUTS)^29^ with a scale range of 0 to 5, respectively.

^c^Measured with the German version of the Patient Health Questionnaire-4 (PHQ-4)^30,31^ with a scale range of 0 to 12.

^d^Classified according to the Global Initiative for Chronic Obstructive Lung Disease (GOLD)^1^.

### Prevalence and characteristics of quit attempts

Of all past-year smokers, 48.5% (*n* = 247, 95% CI 44.2–52.9) reported ≥1 past-year quit attempt. Among this group, 43.3% (*n* = 107) reported a single past-year attempt, 29.1% (*n* = 72) reported two attempts, 14.2% (*n* = 35) reported three attempts, and 13.4% (*n* = 33) reported four or more attempts. Just over half of patients (52.6%, *n* = 130, 95% CI 46.4–58.9) reported that they had attempted to quit by cutting down first, whereas 37.7% (*n* = 93, 95% CI 31.6–43.7) reported that they had stopped abruptly (9.7%, *n* = 24 no answer). Half of the patients (50.2%, *n* = 124, 95% CI 44.0–56.4) reported that their most recent quit attempt was initiated spontaneously, whereas 37.7%, (*n* = 93, 95% CI 31.6–43.7) had planned their attempt in advance (12.1%, *n* = 30 no answer).

### Use of smoking cessation methods

The prevalence of the use of various smoking cessation methods to support the most recent past-year quit attempt is presented in Table 3. From all presented methods, 46.2% (*n* = 114) of the patients had used one method, 22.7% (*n* = 56) had used two methods, 11.3% (*n* = 28) had used three methods, and 6.4% (*n* = 16) had used four or more methods. Around one-third (31.2%, *n* = 77, 95% CI 25.4–37.0) reported the use of ≥1 evidence-based method, of whom 19.0% (*n* = 47, 95% CI 14.1–23.9) used ≥1 behavioural, and 16.2% (*n* = 40, 95% CI 11.6–20.8) used ≥1 pharmacological method. Combined behavioural and pharmacological methods were used by 4.0% (*n* = 10, 95% CI 1.6–6.5).

**Table 3:** Prevalence of the use of evidence-based smoking cessation methods and alternative methods (multiple answers allowed) during the most recent quit attempt among past-year smokers with COPD (FEV1/FVC <0.7) who reported ≥1 past-year quit attempt (*n* = 247).

Smoking cessation method	% (95% CI)
a. Brief physician advice	13.8 (9.5–18.1)
b. Brief advice by a pharmacist	4.9 (2.2–7.5)
c. Behavioural counselling for smoking cessation (Individual or group therapy)	6.1 (3.1–9.1)
d. Behavioural telephone counselling for smoking cessation	0.8 (0.0–1.9)
e. Nicotine replacement therapy (e.g., nicotine patch) with a prescription	4.5 (1.9–7.0)
f. Nicotine replacement therapy (e.g., nicotine patch) without a prescription	10.5 (6.6–14.4)
g. Zyban (Bupropion)	0
h. Champix (Varenicline)	2.0 (0.3–3.9)
i. E-cigarette with nicotine	17.0 (12.3–21.7)
j. E-cigarette without nicotine	7.3 (4.0–10.5)
k. Heated tobacco product (e.g., IQOS or heatsticks)	1.6 (0.0–0.2)
l. Smoking cessation app on a smartphone or tablet computer	0.8 (0.0–1.9)
m. Internet page for smoking cessation	0.8 (0.0–1.9)
n. The book: “Allen Carr’s Easy Way to Stop Smoking”	5.3 (2.5–8.0)
o. Other book on smoking cessation	2.4 (0.5–4.3)
p. Hypnotherapy	1.6 (0.0–3.2)
q. Acupuncture	4.5 (1.9–7.0)
r. Naturopath	1.2 (0.0–2.6)
s. Willpower	42.9 (36.7–49.1)
t. Social environment (family, friends, colleagues)	17.0 (12.3–21.7)
I) ≥1 evidence-based* method (a, c, d, e, f, g and/or h)	31.2 (25.4–37.0)
II) ≥1 evidence-based* behavioural therapeutic method (a, c, and/or d)	19.0 (14.1–23.9)
III) ≥1 evidence-based* pharmacological method (e, f, g and/or h)	16.2 (11.6–20.8)
IV) ≥1 evidence-based* behavioural + pharmacological method (II & III)	4.0 (1.6–6.5)
V) Nicotine replacement therapy with or without prescription (e, f)	14.6 (10.2–19.0)
VI) ≥1 non-evidence-based method (b, i, j, k, l, m, n, o, p, q and/or r)	33.2 (27.3–39.1)
VII) E-cigarette with or without nicotine for quitting (i, j)	21.5 (16.3–26.6)

Data were presented as percentages with 95% CI confidence interval. Data include complete cases.

*Evidence-based = conform to current German clinical practice guidelines for COPD^24,25^.

The most commonly used evidence-based cessation method was NRT with/without prescription (14.6%, *n* = 36, 95% CI 10.2–19.0), followed by brief physician advice (13.8%, *n* = 34, 95% CI 9.5–18.1) and behavioural counselling (individual or group therapy) (6.1%, *n* = 15, 95% CI 3.1–9.1). EC with/without nicotine was the most commonly used method among all investigated methods (21.5%, *n* = 53, 95 % CI 16.3–26.6).

### Characteristics associated with ≥1 past-year quit attempt

Multivariable associations between sociodemographic, smoking, and health-related characteristics of the study sample and the presence of ≥1 past-year quit attempt (= yes) are shown in Table 4. Being male was positively associated with reporting ≥1 past-year quit attempt (OR = 1.50, 95% CI 1.01–2.24), while time spent with urges to smoke was negatively associated with such ≥1 attempt (OR = 0.69 per level on the 6-level scale, 95% CI 0.52–0.91) (see (a) in Table 4). No statistically significant association was found for age, educational qualification, FEV1% predicted, the strength of urges to smoke, and psychological distress. Regressions in the total sample of all 795 past-year smokers listed as COPD patients in the practices showed similar results with the exception that the association with male versus female sex became weaker (see (a) in Supplementary Table 1).

**Table 4:** Multivariable associations between sociodemographic, smoking, and health-related characteristics of past-year smokers with COPD (FEV1/FVC <0.7) and the dichotomous outcomes (a) ≥1 past-year quit attempt (= yes), and (b) use of ≥1 evidence-based smoking cessation method (= yes) during the most recent quit attempt.

Covariates^a^	(a) ≥1 quit attempt (yes vs. no) (imputed *n* = 509)^b^		(b) Use of ≥1 evidence-based cessation method (yes vs. no) (imputed *n* = 247)^c^	
	OR (95% CI)	*p* value	OR (95% CI)	*p* value
Age^¥^	0.99 (0.97–1.02)	0.598	0.99 (0.95–1.02)	0.402
Sex				
Female (Reference)	1		1	
Male	1.50 (1.01–2.24)	0.046	1.32 (0.73–2.40)	0.358
Educational qualification^d^				
Low (Reference)	1		1	
Medium	0.81 (0.51–1.30)	0.378	1.46 (0.70–3.05)	0.309
High	1.12 (0.60–2.08)	0.727	1.56 (0.64–3.79)	0.325
Time spent with urges to smoke^e¥^	0.69 (0.52–0.91)	0.011	0.89 (0.61–1.30)	0.543
Strength of urges to smoke^e¥^	0.93 (0.70–1.24)	0.632	1.62 (1.09–2.41)	0.018
Psychological distress^f¥^	1.07 (0.99–1.15)	0.105	1.00 (0.91–1.10)	0.978
FEV1% predicted^g¥^	0.99 (0.98–1.00)	0.196	1.00 (0.99–1.02)	0.722

Data were presented as odds ratios (OR) and 95% confidence interval (CI).

^a^Analyses were adjusted for all listed covariates.

^b^Past-year smokers with COPD (FEV1/FVC <0.7); multiple imputation was used to impute the missing values for educational qualification, time spent with urges to smoke, the strength of urges to smoke, psychological distress, and quit attempts.

^c^Past-year smokers with COPD (FEV1/FVC <0.7) who reported ≥1 past-year quit attempt; multiple imputation was used to impute the missing values for educational qualification, time spent with urges to smoke, the strength of urges to smoke, and psychological distress.

^d^German educational qualification: low = 9 years of education or no graduation, medium = 10 years of education, high = ≥11 years of education.

^e^Both items were measured with the German version of the Strength of Urges to Smoke Scale (SUTS)^29^ with a scale range of 0 to 5, respectively.

^f^Measured with the German version of the Patient Health Questionnaire-4 (PHQ-4)^30,31^ with a scale range of 0 to 12.

^g^FEV1 forced expiratory volume in 1 s; % predicted: percentage of the predicted value.

^¥^Continuous variable.

### Characteristics associated with the use of ≥1 evidence-based smoking cessation method

Multivariable associations between sociodemographic, smoking and health-related characteristics of the study sample and the use of ≥1 evidence-based smoking cessation method during the most recent past-year quit attempt are shown in Table 4. The strength of urges to smoke was positively associated with the use of ≥1 evidence-based smoking cessation method (OR = 1.62 per level on the six-level scale, 95% CI 1.09–2.41) (see (b) in Table 4). No statistically significant association was found for age, sex, educational qualification, FEV1% predicted, time spent with urges to smoke, and psychological distress. Regressions in the total sample of all 795 past-year smokers listed as COPD patients in the practices yielded similar results (see (b) in Supplementary Table 1).

### Ancillary analysis of current motivation to stop smoking

In the total of 416 current smokers with COPD (FEV1/FVC <0.70), 2.6% did not answer the question, 33.4% (*n* = 139, 95% CI 28.9–37.9) was unmotivated to quit (response 1 and 2), and 64.0% (*n* = 266, 95% CI 59.3–68.6) was motivated to stop smoking (response 3–7), including 15.7% of those with a clear intention to do so in the next 1 to 3 months (response 6 and 7) (see Supplementary Table 2).

## Discussion

Among adult past-year smokers with spirometry-confirmed COPD from pulmonary practices in Germany, almost every second patient reported at least one past-year attempt to quit tobacco smoking. The most recent quit attempt was made rather by cutting down first and spontaneously. One-third of patients reported the use of at least one evidence-based method to support their most recent past-year quit attempt. Only 4% of attempts were supported by a combination of behavioural and pharmacological treatments. EC was the most commonly used cessation method. Male sex was positive, and time spent with urges to smoke was negatively associated with reporting at least one past-year quit attempt. The strength of urges to smoke was positively associated with the use of at least one evidence-based smoking cessation method.

The prevalence of past-year quit attempts among adult smokers with COPD determined in our study is comparable to two former international population-based household surveys (Canada: 48%^20^; US: 52%^21^), whereas two recent population-based telephone surveys from the US reported slightly higher rates of 60–65%^22,23^. However, it is notable to mention that the prevalence of quit attempts in the general smoking population is already higher in the US compared with data from Germany (56–57%^37^ versus 19%^28^).

Compared with smokers of the general population in Germany, the prevalence of reporting a past-year quit attempt determined among the COPD patients in our study is substantially higher (49 versus 19%^28^), and more current smokers with COPD want to stop smoking (64.0 versus 38.8%^35^). In a population-based study from the US, quit attempt rates among smokers with COPD were also significantly higher than in smokers without COPD^21^. These results suggest that smokers with COPD are highly motivated to quit harmful tobacco smoking.

Evidence on associations between individual characteristics and quit attempts in smokers with COPD is ambiguous. While our study found a positive association with being male and a negative association with time spent with urges to smoke, international studies reported positive associations with younger age^21^, female gender^22^ and higher educational qualification^23^. However, it is notable to mention methodological differences between studies, such as the use of partially different exposure variables.

The association between increasing time spent with urges to smoke and less odds of quit attempts shown in our study may be explained by the fact that tobacco dependence reduces self-efficacy^13,38^, which plays an important role in smoking cessation^39^. Thus, smokers with COPD who feel constant urges to smoke probably tend not to attempt to quit harmful smoking because they don’t believe in their ability to stop.

The usage of evidence-based smoking cessation methods determined in our study is substantially lower than in international COPD populations^17,23^, and only a small fraction of smokers with COPD (4%) report the use of combined behavioural and pharmacological treatments as recommended in the German COPD guidelines^24,25^. This underuse is probably because this population does not receive adequate information about evidence-based smoking cessation methods and sufficient advice to use them^40,41^. Smokers with lung conditions not only need clear advice to quit but also extensive and target-group-specific information on effective treatment options^26^. However, health professionals in Germany experience a lack of training in how to deliver such smoking cessation counselling effectively and efficiently to their patients^42^, as such training is not standard in undergraduate and postgraduate medical training^43^. In addition, there are other, more structural deficits in the German healthcare system, including the lack of reimbursement of evidence-based smoking cessation methods and the lack of availability of professional smoking cessation services across the country^40^, although such access is particularly important for smokers with lung conditions^26^. The lack of reimbursements of costs for important therapies may represent a huge financial barrier towards the use of such methods, particularly towards the use of combined behavioural and pharmacological treatments. The fact that patients seem to underestimate the effectiveness of evidence-based smoking cessation methods^44^ may also be a barrier to the use of these treatments.

Compared with past-year smokers from the German general population, about twice as many smokers with COPD in our study reported the use of evidence-based smoking cessation methods during the most recent past-year quit attempt (31 versus 13%^28^). Comparable results were found in studies from the Netherlands and the US when assessing the ever use of these methods^17^ and the use during the last quit attempt^23^ among smokers with and without COPD. Previous population-based studies from Germany and England found an increase in the use of evidence-based smoking cessation methods with increasing levels of tobacco dependence^28,45^. Probably, smokers with greater tobacco dependence and associated withdrawal symptoms experience greater difficulties when quitting without any assistance and are therefore more likely to seek support^28^. In our study, increasing the strength of urges to smoke was comparably associated with higher odds of the use of such methods. Considering the fact that smokers with COPD already have a higher level of tobacco dependence compared with other smokers^15–17^, higher usage rates of evidence-based smoking cessation methods among the COPD population are as expected.

A relatively large number of smokers with COPD in our study (22%) reported the use of EC to support their most recent past-year quit attempt, and the usage was about twice as high as in the German general smoking population^28^. Although the effectiveness of EC for smoking cessation in smokers with COPD is still discussed^24,25^, previous findings suggest that the use of EC helps smokers with COPD to reduce cigarette consumption or prevent relapse^46^.

Our study provides detailed data on quit attempts and the use of smoking cessation methods, as well as data on associations with sociodemographic, smoking, and health-related characteristics in patients with COPD in a pulmonary primary care setting in Germany. As data collection took place in this setting, it was possible to recruit a broad patient collective, which included both routine control patients and ‘emergency’ patients who had current symptoms of (respiratory) infection and/or mild or moderate exacerbation. Data on lung function parameters of patients were delivered by the practices and are therefore less error-prone and more reliable than patients’ self-reports. Based on this data, we included only patients with a post-bronchodilator ratio of FEV1/FVC <0.70 and thus only patients with a valid COPD diagnosis according to the most recent GOLD guidelines^1^.

However, all other data were self-reported, increasing the risk of missing data. Some variables of interest in our study contained missingness, and it remained unclear if respondents skipped questions intentionally, by mistake, or because of an inability to provide an answer. Missing data were therefore imputed. Data on quit attempts were collected retrospectively, increasing the risk of recall bias that may have affected the prevalence estimates, as short-lasting or occurring further in the past quit attempts may fail to be reported^47^. Moreover, due to the conservative inclusion criterion of a post-bronchodilator ratio of FEV1/FVC <0.70, a relatively high number of past-year smokers (36%) were excluded from our analyses. This led to a relatively small sample size in our study, and the statistical power was probably too low to detect meaningful associations between exposure variables and determined outcomes. Furthermore, our data did not allow an investigation of adherence to smoking cessation methods.

In conclusion, around every second smoking patient with COPD in the German pulmonary primary care setting reports a past-year quit attempt, mainly independent of individual sociodemographic or health-related characteristics. Quit attempts are rarely supported by evidence-based smoking cessation methods, and hardly ever under the application of combined behavioural and pharmacological treatments, although recommended in COPD Guidelines. Urges to smoke seem to play an important role in attempting to quit and using evidence-based methods. EC is the most commonly used cessation method, although the effectiveness and safety of EC as a cessation method should be further investigated among smokers with COPD who do not want to use recommended evidence-based treatments. These quitting characteristics should be taken into account by physicians while helping their COPD patients to quit harmful smoking.

## Supplementary information

Supplementary Tables

REPORTING SUMMARY

## Data Availability

The data underlying this study are available to researchers from the corresponding author (Sabrina.Kastaun@med.uni-duesseldorf.de). All proposals requesting data access will need to specify how it is planned to use the data, and all proposals will need approval from the RESPIRO study team before the data release.

## Code availability

The code (i.e. SPSS syntax) for the statistical analyses is available to researchers from the corresponding author (Sabrina.Kastaun@med.uni-duesseldorf.de).
