# Supplementary material for: Attempts to quit smoking, use of smoking cessation methods, and associated characteristics among COPD patients
Source: NPJ Prim Care Respir Med. 2022 Nov 10;32:50. doi: 10.1038/s41533-022-00316-5 (PMC9646777; doi:10.1038/s41533-022-00316-5)
Supplement: Supplementary file 1 — Supplementary Tables [file 41533_2022_316_MOESM1_ESM.pdf]

Supplementary Table 1. Multivariable associations between sociodemographic, smoking, and health-related characteristics of past-year smokers listed as COPD patients in the practices and the dichotomous outcomes (a) ≥1 past-year quit attempt (=yes), and (b) use of ≥1 evidence-based smoking cessation method (=yes) during the most recent quit attempt.

Covariates[a]	(a) ≥1 quit attempt (yes vs. no) (imputed *n*=795)[b]		(b) Use of ≥1 evidence-based cessation method (yes vs. no) (imputed *n*=391)[c]	
	OR (95% CI)	*p*-value	OR (95% CI)	*p*-value
Age[¥]	0.99 (0.97 – 1.01)	0.308	0.99 (0.96 – 1.02)	0.446
Sex				
Female (Reference)	1		1	
Male	1.21 (0.88 – 1.66)	0.236	1.19 (0.76 – 1.86)	0.458
Educational qualifications[d]				
Low (Reference)	1		1	
Medium	0.71 (0.47 – 1.06)	0.094	1.34 (0.73 – 2.48)	0.344
High	0.98 (0.59 – 1.62)	0.929	1.32 (0.66 – 2.73)	0.424
Time spent with urges to smoke[e¥]	0.68 (0.56 – 0.84)	<0.001	0.94 (0.71 – 1.23)	0.633
Strength of urges to smoke[e¥]	0.93 (0.72 – 1.21)	0.593	1.50 (1.12 – 2.01)	0.007
Psychological distress[f¥]	1.06 (1.00 – 1.12)	0.059	1.02 (0.94 – 1.10)	0.659
FEV1% predicted[g¥]	0.99 (0.98 – 1.00)	0.105	1.01 (1.00 – 1.02)	0.167

Note: Data are presented as Odds Ratios (OR) and 95% confidence interval (CI).

[a] Analyses were adjusted for all listed covariates.

[b] Past-year smokers listed as COPD patients in the practices according to ICD-10[27] (including those with FEV1/FVC≥0.7); multiple imputation was used to impute the missing values for age, sex, educational qualification, time spent with urges to smoke, strength of urges to smoke, psychological distress, and quit attempts.

[c] Past-year smokers listed as COPD patients in the practices according to ICD-10[27] (including those with FEV1/FVC≥0.7) who reported ≥1 past-year quit attempt; multiple imputation was used to impute the missing values for educational qualification, time spent with urges to smoke, strength of urges to smoke, and psychological distress.

[d] German educational qualification: low=9 years of education or no graduation, medium=10 years of education, high=≥11 years of education.

[e] Both items measured with the German version of the Strength of Urges to Smoke Scale (SUTS)[29] with a scale range of 0 to 5, respectively.

[f] Measured with the German version of the Patient Health Questionnaire-4 (PHQ-4)[30,31] with a scale range of 0 to 12.

[g] FEV1: forced expiratory volume in 1 second; % predicted: percentage of the predicted value.

[¥] Continuous variable.

Supplementary Table 2. Current motivation to stop smoking among 416 current smokers with COPD (FEV1/FVC<0.7).

Motivation to stop smoking[a]<br>*Which of the following describes you?*	Total<br>% (n)
1: "I don't want to stop smoking"	4.6 (19)
2: "I think I should stop smoking but don't really want to"	28.8 (120)
3: "I want to stop smoking but haven't thought about when"	8.2 (34)
4: "I REALLY want to stop smoking but I don't know when I will"	12.0 (50)
5: "I want to stop smoking and hope soon"	28.1 (117)
6: "I REALLY want to stop smoking and intend to in the next 3 months"	8.2 (34)
7: "I REALLY want to stop smoking and intend to in the next month"	7.5 (31)

Note: Data are presented as percentage (number, *n*). Difference when calculating the total percentage can be explained by missing data on the variable.
[a] Measured with the German version of the Motivation To Stop Scale (MTSS)[35,36].
