# Supplementary material for: Attempts to quit smoking, use of smoking cessation methods, and associated characteristics among COPD patients
Source: NPJ Prim Care Respir Med. 2022 Nov 10;32:50. doi: 10.1038/s41533-022-00316-5 (PMC9646777; doi:10.1038/s41533-022-00316-5)
Supplement: Supplementary file 2 — REPORTING SUMMARY [file 41533_2022_316_MOESM2_ESM.pdf]

nature portfolio

Corresponding author(s): Dr. Sabrina Kastaun
NPJPCRM-01597R

Last updated by author(s): Sep 11, 2022

Reporting Summary

Nature Portfolio wishes to improve the reproducibility of the work that we publish. This form provides structure for consistency and transparency in reporting. For further information on Nature Portfolio policies, see our Editorial Policies and the Editorial Policy Checklist.

Statistics

For all statistical analyses, confirm that the following items are present in the figure legend, table legend, main text, or Methods section.

n/a	Confirmed	
☐	☒	The exact sample size (*n*) for each experimental group/condition, given as a discrete number and unit of measurement
☐	☒	A statement on whether measurements were taken from distinct samples or whether the same sample was measured repeatedly
☐	☒	The statistical test(s) used AND whether they are one- or two-sided<br>*Only common tests should be described solely by name; describe more complex techniques in the Methods section.*
☐	☒	A description of all covariates tested
☐	☒	A description of any assumptions or corrections, such as tests of normality and adjustment for multiple comparisons
☐	☒	A full description of the statistical parameters including central tendency (e.g. means) or other basic estimates (e.g. regression coefficient) AND variation (e.g. standard deviation) or associated estimates of uncertainty (e.g. confidence intervals)
☐	☒	For null hypothesis testing, the test statistic (e.g. *F*, *t*, *r*) with confidence intervals, effect sizes, degrees of freedom and *P* value noted<br>*Give P values as exact values whenever suitable.*
☒	☐	For Bayesian analysis, information on the choice of priors and Markov chain Monte Carlo settings
☒	☐	For hierarchical and complex designs, identification of the appropriate level for tests and full reporting of outcomes
☒	☐	Estimates of effect sizes (e.g. Cohen's *d*, Pearson's *r*), indicating how they were calculated

Our web collection on statistics for biologists contains articles on many of the points above.

Software and code

Policy information about availability of computer code

Data collection	Paper questionnaire, Data entry: IBM SPSS Statistics Version 28.0
Data analysis	IBM SPSS Statistics Version 28.0

For manuscripts utilizing custom algorithms or software that are central to the research but not yet described in published literature, software must be made available to editors and reviewers. We strongly encourage code deposition in a community repository (e.g. GitHub). See the Nature Portfolio guidelines for submitting code & software for further information.

Data

Policy information about availability of data

All manuscripts must include a data availability statement. This statement should provide the following information, where applicable:

- Accession codes, unique identifiers, or web links for publicly available datasets
- A description of any restrictions on data availability
- For clinical datasets or third party data, please ensure that the statement adheres to our policy

The data underlying this study are available to researchers on reasonable request from the corresponding author (Sabrina.Kastaun@med.uni-duesseldorf.de). All proposals requesting data with the purpose to explore own research ideas access will need to specify how it is planned to use the data, and all proposals will need approval of the RESPIRO study team prior to data release.

Human research participants

Policy information about studies involving human research participants and Sex and Gender in Research.

Reporting on sex and gender	Findings apply to sex (female and male). Sex was determined based on participants self-reporting. Regression analyses were analysed by using sex as a covariate.
Population characteristics	Cross-sectional survey among patients with a spirometry-confirmed diagnosis of COPD (ICD-10: J44.x) aged ≥18 from pulmonary practices across the German federal state of North Rhine-Westphalia (NRW).
Recruitment	Pulmonary practices were recruited through the scientific information network of the Scientific Institute for Health Care Research in Pneumology "WINPNEU" (http://winpneu.de), initiated by the German federal association of pulmonologists, sleep and respiratory physicians.<br>A contact person within the practice (i.e. the pulmonologist, or a study nurse) carried out the recruitment of patients. Eligible patients received all study materials (questionnaire, informed consent form, and a small non-financial incentive) to take it home with them. They were asked to read the study information carefully and to send the completed documents back to the study centre, if they decided to participate in the study.<br>Such a recruitment may lead to potential self-selection bias. For example, particularly motivated persons may be more likely to participate in the survey, while those feeling unwell or very sick as a result of their disease may be less likely to participate in the survey. This bias may potentially affect the representativeness of the investigated sample. However, the number of study participants is reported in the manuscript (see Table 2) stratified by GOLD stages of the disease (severity of COPD) and it shows that around 50% of the patients have mild or moderate symptoms of COPD while the other half has severe or very severe symptoms. The prevalence of tobacco smoking in our overall COPD patient sample was somewhat higher than in the general German population (40% vs. 30%), which is also very plausible. We thus assume a relatively good representativeness of our sample to the clinical population of COPD patients in German pulmonary practices.
Ethics oversight	The RESPIRO study was approved by the ethics committee of the Medical Faculty of the Heinrich-Heine-University Duesseldorf, Germany (ID 5680R).

Note that full information on the approval of the study protocol must also be provided in the manuscript.

Field-specific reporting

Please select the one below that is the best fit for your research. If you are not sure, read the appropriate sections before making your selection.

☐ Life sciences ☒ Behavioural & social sciences ☐ Ecological, evolutionary & environmental sciences

For a reference copy of the document with all sections, see nature.com/documents/nr-reporting-summary-flat.pdf

Behavioural & social sciences study design

All studies must disclose on these points even when the disclosure is negative.

Study description	Quantitative cross-sectional survey.
Research sample	509 past-year smokers (81.7% = current smokers and 18.3% = recent ex-smokers (≤12 months since quitting)) with a spirometry-confirmed diagnosis of COPD (post-bronchodilator ratio of FEV1/FVC<0.70) from 19 pulmonary practices across the German federal state of NRW.<br>The sample included 43.8% (n=223) female patients and 56.% (n=286) male patients. The mean age was 62.8 years (SD = 8.4, range 35-87 years). Of a total sample, 55.8% (n = 284) had no/a low level of education, 22.6% (n = 115) had a medium level of education secondary school leaving certificate (grade ten)) and only 11.6% (n = 59) had a high level of education (general higher education entrance qualification/ high school diploma).<br>This research sample was chosen because the aim of our study was to explore past-year quit attempts, cessation methods used, and associations with person characteristics among smokers with spirometry-confirmed COPD diagnosis according to the Global Initiative for Chronic Obstructive Lung Disease (GOLD).<br><br>Recruitment may have lead to potential self-selection bias. For example, particularly motivated persons may be more likely to participate in the survey, while those feeling unwell or very sick as a result of their disease may be less likely to participate in the survey. Such bias may have potentially affected the representativeness of the invetigated sample. However, the number of study participants is reported in the manuscript (see Table 2) stratified by GOLD stages of the disease (severity of COPD) and it shows that around 50% of the patients have mild or moderate symptoms of COPD while the other half has severe or very severe symptoms. The prevalence of tobacco smoking in our overall COPD patient sample was somewhat higher than in the general German population (40% vs. 30%), which is also very plausible. We thus assume a relatively good representativeness of our sample to the clinical population of COPD patients in German pulmonary practices.
Sampling strategy	For this explorative survey, no specific sample-size calculation was performed. We expected to rectruit about 20 to 25 pulmonary practices to participate in our study during the study period. Within these practices we assumed that at least 35% were current

tobacco smokers or recent ex-smokers, and we assumed a participation rate of patients of ~30-35%. These numbers were based on former studies in COPD patients in Germany, as well as on own experiences with studies in smokers in German primary care. Our explorative regression analyses adhered to the well-used and often published "rule of thumb" for sample size of at least 10 participants per predictor variable.
Regarding the sampling startegy, we asked the responsable study nurse of each participating practice or the pneumologist to hand out the study material consecutively to every patient presenting with the inclusion criteria during the study period. We cannot rule out that nurses/pneumogogists selectively included or excluded specific patients (due to the patients' physical or emotional burden, or due to own time constraints). This may have affected the representativeness of our sample. However, the number of study participants is reported in the manuscript (see Table 2) stratified by GOLD stages of the disease (severity of COPD) and it shows that around 50% of the patients have mild or moderate symptoms of COPD while the other half has severe or very severe symptoms. The prevalence of tobacco smoking in our overall COPD patient sample was somewhat higher than in the general German population (40% vs. 30%), which is also very plausible. We thus assume a relatively good representativeness of our sample to the clinical population of COPD patients in German pulmonary practices.

Data collection

Research data were collected via a pen and paper questionnaire.
At first step, the contact person within the practice (i.e. the pulmonologist, or a study nurse), which carried out the recruitment of eligible patients, also documented clinical characteristics of participating patients in the study questionnaire, including the ICD-10 COPD diagnosis code and lung function parameters of the most recent spirometry: forced expiratory volume in 1 second (FEV1), forced vital capacity (FVC), and FEV1% predicted.
At second step, recruted patients received the questionnaire from the contakt person to take it home with them and were asked to send the completed questionnaire back to the study centre.
Data collected were:
- Sociodemographic characteristics: age, sex (female, male), edicational qualification (low=9 years of education or no graduation, medium=10 years, high≥11 years),
- Smoking status: current smokers of cigarettes or other combustible tobacco products (e.g., pipe, cigars), recent ex-smokers (≤12 months since quitting), long-term ex-smokers (>12 months since quitting), and never smokers
- Time spent with urges to smoke and strength of urges to smoke,
- Psychological distress: symptoms of a major depression and generalized anxiety,
- Past-year quit attempts,
- The use of evidence-based and alternative smoking cessation methods during the most recent quit attempt,
- Additionally: current motivation to stop smoking of current smokers.

Timing

Data collection took place between September 2018 and June 2020.

Data exclusions

For our research, we conservatively included only past-year smokers with a post-bronchodilator ratio of FEV1/FVC<0.70, which we calculated according to the Global Initiative for Chronic Obstructive Lung Disease (GOLD) criteria for the diagnosis of COPD.
Of all 795 past-year smokers listed as ICD-10 COPD patients in the practices, 286 (36%) had a FEV1/FVC≥0.70 and were thus excluded from the analyses. This resulted in a final study sample of 509 past-year smokers with a spirometry-confirmed COPD diagnosis.

Non-participation

Within the scope of the RESPIRO study, of 4377 distributed questionnaires, 2012 questionnaires were sent back. The study had thus a response rate of 46%. Six (0.3%) responders withdrew their informed consent to participate afterwards.

Randomization

N/A.

Reporting for specific materials, systems and methods

We require information from authors about some types of materials, experimental systems and methods used in many studies. Here, indicate whether each material, system or method listed is relevant to your study. If you are not sure if a list item applies to your research, read the appropriate section before selecting a response.

Materials & experimental systems

n/a	Involved in the study	
☒	☐	Antibodies
☒	☐	Eukaryotic cell lines
☒	☐	Palaeontology and archaeology
☒	☐	Animals and other organisms
☐	☒	Clinical data
☒	☐	Dual use research of concern

Methods

n/a	Involved in the study	
☒	☐	ChIP-seq
☒	☐	Flow cytometry
☒	☐	MRI-based neuroimaging

Clinical data

Policy information about clinical studies
All manuscripts should comply with the ICMJE guidelines for publication of clinical research and a completed CONSORT checklist must be included with all submissions.

Clinical trial registration

DRKS00015450

Study protocol

Analysis plan: https://osf.io/a24t3/

Data collection

Data collection took place in pulmonary practices across the German federal state of North Rhine-Westphalia (NRW). The first practice was recruited in September 2018 and the last one in July 2019. The first patient was included in September 2018 and recruitment of the patients took place until the end of April 2020. Data collection occurred till June 2020.

Outcomes

Outcome measures:
1) Past-year quit attempts were measured by asking: "How many serious attempts to stop smoking have you made in the last 12 months? By serious attempt I mean you decided that you would try to make sure you never smoked again. Please include any attempt that you are currently making and please include any successful attempt made within the last year."
2) The use of evidence-based and alternative smoking cessation methods during the most recent quit attempt was measured by asking: "Which, if any, of the following did you try to help you stop smokingduring the most recent serious quit attempt? (Multiple answers allowed):
a. Brief physician advice
b. Brief advice by a pharmacist
c. Behavioural counselling for smoking cessation (Individual or group therapy)
d. Behavioural telephone counselling for smoking cessation
e. Nicotine replacement therapy (e.g., nicotine patch) with a prescription
f. Nicotine replacement therapy (e.g., nicotine patch) without a prescription
g. Zyban (Bupropion)
h. Champix (Varenicline)
i. E-cigarette with nicotine
j. E-cigarette without nicotine
k. Heated tobacco product (e.g., IQOS or heatsticks)
l. Smoking cessation app on a smartphone or tablet computer
m. Internet page for smoking cessation
n. The book: “Allen Carr’s Easy Way to Stop Smoking”
o. Other book on smoking cessation
p. Hypnotherapy
q. Acupuncture
r. Naturopath
s. Willpower
t. Social environment (family, friends, colleagues)

Evidence-based methods were chosen according to the current German guidelines on the treatment of tobacco addiction in COPD patients: behavioural (brief physician advice, individual, group or telephone counselling) and pharmacological (NRT with/without a prescription, varenicline, and bupropion) treatments. For regression analyses, we coded a new dichotomous variable “use of ≥1 evidence-based method” (yes versus no). Alternative methods were chosen according to frequently used methods in the German general smoking population.
